# Inhibition kinetics of *Plasmodium* Lactate Dehydrogenase with herbal extracts suggest possible enzyme inhibitor molecular interaction

**DOI:** 10.1186/1475-2875-11-S1-P56

**Published:** 2012-10-15

**Authors:** Privadarshan Keluskar, Apeksha Mohile, Sanjay Ingle

**Affiliations:** 1 Department of Microbiology, Faculty of Science, M. S. University of Baroda, Vadodara, Gujarat, 390002, India

## Background

Resistance acquired by *Plasmodium* species (especially *P. falciparum*) to most of the present antimalarial drugs is the principal hindrance in controlling malaria. Thus, one of the major challenges towards elimination of Malaria is development of novel and sustainable antimalarial drugs. In this milieu, herbs traditionally used to treat malaria are promising repertoires of antimalarial drugs. Earlier, lab studies have reported selective inhibition of *P. falciparum* and *P. vivax* specific Lactate Dehydrogenase (PfLDH and PvLDH) by *Phyllanthus amarus* aqueous extract and *Murraya koenigii* chloroform extract respectively. In the present investigation, we studied inhibition kinetics of PfLDH and PvLDH to explore molecular interactions between enzyme and inhibitor.

## Materials and methods

Recombinant PfLDH and PvLDH, expressed in *E. coli*, were used in enzyme assay. LDH activity was measured in the direction of pyruvate to L-lactate conversion[1]. Steady state kinetic constants for substrate and cofactor as well as inhibition constants for plant extracts were measured by double reciprocal plot (Lineweaver and Burk plot). The enzyme inhibitor interactions were determined based on variations in the kinetic constants in presence of inhibitors, compared to control.

## Results

Enzyme inhibition kinetics results are summarised in Table 1.

**Table 1:** 

		**NADH (Cofactor)**		**Pyruvate (Substrate)**	
		
**Plant Extracts**	**Enzymes**	**Type of Inhibition**	**Inhibition constant K_i_(µg/ml)**	**Type of Inhibition**	**Inhibition constant K_i_ (µg/ml)**
*P. amarus* aqueous extract	PfLDH	Competitive	4.1±0.7	Noncompetitive	9.6±1.8
	PvLDH	Competitive	1.9±0.2	Competitive	3.5±0.2
*M. koenigii* chloroform extract	PfLDH	Noncompetitive	2.6±0.7	Competitive	1.1±0.3
	PvLDH	Noncompetitive	2.3±0.4	Linear Mixed	0.8±0.2

## Conclusion

*P. amarus* aqueous extracts contain PfLDH and PvLDH inhibitors, interacting at cofactor binding site. *M. koenigii* chloroform extracts contain PfLDH inhibitors, interacting at substrate binding site and PvLDH inhibitors, interacting at cofactor binding site. As parasite LDH inhibitors with affinity for cofactor binding site, have significant therapeutic value[2]; our studies have confirmed the importance of potential antimalarial compounds present in studied extracts and further investigation may lead to development of target specific antimalarial drugs.
